# Safety, tolerability and appropriate use of nintedanib in idiopathic pulmonary fibrosis

**DOI:** 10.1186/s12931-015-0276-5

**Published:** 2015-09-24

**Authors:** Tamera Corte, Francesco Bonella, Bruno Crestani, Maurits G. Demedts, Luca Richeldi, Carl Coeck, Katy Pelling, Manuel Quaresma, Joseph A. Lasky

**Affiliations:** Royal Prince Alfred Hospital, Camperdown, New South Wales Australia; University of Sydney, Sydney, New South Wales Australia; Ruhrlandklinik, University Hospital, University of Duisburg-Essen, Essen, Germany; Hôpital Bichat, Pneumologie, Paris, France; University Hospital Leuven, Leuven, Belgium; National Institute for Health Research Southampton Respiratory Biomedical Research Unit and Clinical and Experimental Sciences, University of Southampton, Southampton, UK; SCS Boehringer Ingelheim Comm.V., Brussels, Belgium; Boehringer Ingelheim Ltd., Bracknell, UK; Boehringer Ingelheim Pharma GmbH & Co. KG, Ingelheim am Rhein, Germany; Tulane University School of Medicine, New Orleans, LA USA

## Abstract

**Background:**

Idiopathic pulmonary fibrosis (IPF) is a progressive disease characterised by dyspnea and loss of lung function.

**Methods:**

Using pooled data from the replicate, randomized, 52-week, placebo-controlled INPULSIS^®^ trials, we characterized the safety and tolerability of nintedanib 150 mg twice daily in patients with IPF and described how adverse events were managed during these trials.

**Results:**

One thousand and sixty- one patients were treated (nintedanib 638; placebo 423). Higher proportions of patients in the nintedanib group than the placebo group had ≥1 dose reduction to 100 mg bid (27.9 % versus 3.8 %) or treatment interruption (23.7 % versus 9.9 %). Adverse events led to permanent treatment discontinuation in 19.3 % and 13.0 % of patients in the nintedanib and placebo groups, respectively. Diarrhea was the most frequent adverse event, reported in 62.4 % of patients in the nintedanib group versus 18.4 % in the placebo group; however, only 4.4 % of nintedanib-treated patients discontinued trial medication prematurely due to diarrhea. Monitoring of liver enzymes before and periodically during nintedanib treatment was recommended so that liver enzyme elevations could be managed through dose reduction or treatment interruption.

**Conclusion:**

Nintedanib had a manageable safety and tolerability profile in patients with IPF. Recommendations for adverse event management minimized permanent treatment discontinuations in the INPULSIS^®^ trials.

**Trial registration:**

clinicaltrials.gov NCT01335464 and NCT01335477

**Electronic supplementary material:**

The online version of this article (doi:10.1186/s12931-015-0276-5) contains supplementary material, which is available to authorized users.

## Background

Idiopathic pulmonary fibrosis (IPF) is a progressive, fibrosing interstitial pneumonia, occurring primarily in the elderly population, which is characterized by increasing dyspnea and loss of lung function [1–3]. Patients with IPF have impaired quality of life and reduced exercise tolerance [4]. IPF is ultimately fatal, with a median survival time of only 2–3 years from diagnosis [3].

Our evolving understanding of the pathophysiology of IPF suggests complex interplay between multiple pathways. Protein tyrosine kinases are known to play a key role in intracellular signaling pathways involved in the pathogenesis of lung fibrosis [5–7]. Nintedanib is an intracellular inhibitor of tyrosine kinases, including the receptors for the fibroblast growth factor (FGF), platelet-derived growth factor (PDGF) and vascular endothelial growth factor (VEGF) [8, 9]. In preclinical studies nintedanib interfered with processes associated with fibrosis including fibroblast proliferation, migration and differentiation and was associated with reduced secretion of extracellular matrix and reduced lung inflammation [9].

The efficacy and safety of nintedanib as a treatment for IPF have been studied in the Phase II, 52-week, TOMORROW trial [10] and in the two replicate randomized, placebo-controlled, 52-week Phase III INPULSIS^®^ trials [11, 12]. Results from the TOMORROW trial, which involved 432 patients with IPF, suggested that compared with placebo, treatment with nintedanib 150 mg twice daily (bid) was associated with a reduced annual decline in forced vital capacity (FVC), fewer acute exacerbations, and preservation of health-related quality of life, assessed using the St George’s Respiratory Questionnaire (SGRQ) [10]. In both INPULSIS^®^ trials, the primary endpoint of the annual rate of decline in FVC was significantly reduced by approximately 50 % in the nintedanib group compared with placebo, consistent with a slowing of disease progression. The adjusted annual rate of decline in FVC was −114.7 mL/year with nintedanib versus −239.9 mL/year with placebo (a difference of 125.3 mL/year [95 % confidence interval (CI): 77.7, 172.8]; *p* < 0.001) in INPULSIS^®^-1 and −113.6 mL/year with nintedanib versus −207.3 mL/year with placebo (a difference of 93.7 mL/year [95 % CI: 44.8, 142.7]; *p* < 0.001) in INPULSIS^®^-2 [11]. In INPULSIS^®^-2, there was a significant difference in favor of nintedanib for time to first acute exacerbation and change from baseline in SGRQ total score, both over 52 weeks. However, in INPULSIS^®^-1, no significant difference between groups was observed for either of these endpoints [11]. Gastrointestinal events, particularly diarrhea, were the most frequently reported adverse events in patients treated with nintedanib.

An analysis of pooled safety data from the two INPULSIS^®^ trials was pre-specified. In this manuscript, we further characterize the safety and tolerability of nintedanib and describe how adverse events were reported and managed during these Phase III trials.

## Methods

The INPULSIS^®^ trials, performed at 205 sites in 24 countries in the Americas, Europe, Asia and Australia, recruited a broad range of patients with IPF [11]. Patients with IPF aged ≥40 years were eligible to participate if they had an FVC of ≥50 % of predicted value and a diffusing capacity of the lung for carbon monoxide (DL_CO_) of 30–79 % of predicted value. In the absence of a surgical lung biopsy, diagnosis of IPF required the presence of honeycombing and/or a combination of traction bronchiectasis and reticulation, without nodules or consolidation, on high-resolution computed tomography (HRCT), which was centrally reviewed. If available, surgical lung biopsies were centrally reviewed to confirm eligibility to enter the trials using a multidisciplinary approach. Patients with emphysema on HRCT were not excluded, but patients were required to have an FEV_1_/FVC ratio of ≥0.7. Concomitant therapy with prednisone ≤15 mg/day, or the equivalent, was permitted if the dose had been stable for ≥8 weeks before screening. Patients receiving other therapies for IPF, including pirfenidone, prednisone >15 mg/day, azathioprine, N-acetylcysteine and any investigational treatments for IPF, were excluded. Patients with elevated hepatic enzymes (alanine aminotransferase [ALT] or aspartate aminotransferase [AST]) or bilirubin >1.5 x upper limit of normal (ULN) were excluded.

**Fig. 1:**
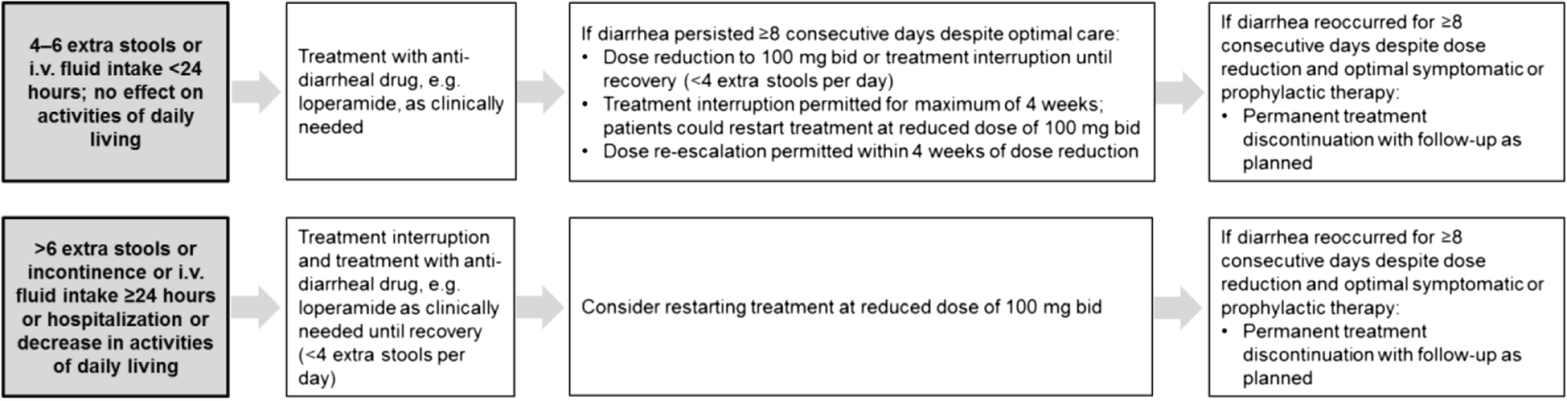
Algorithm for the management of diarrhea adverse events in the INPULSIS^®^ trials. i.v., intravenous

**Fig. 2:**
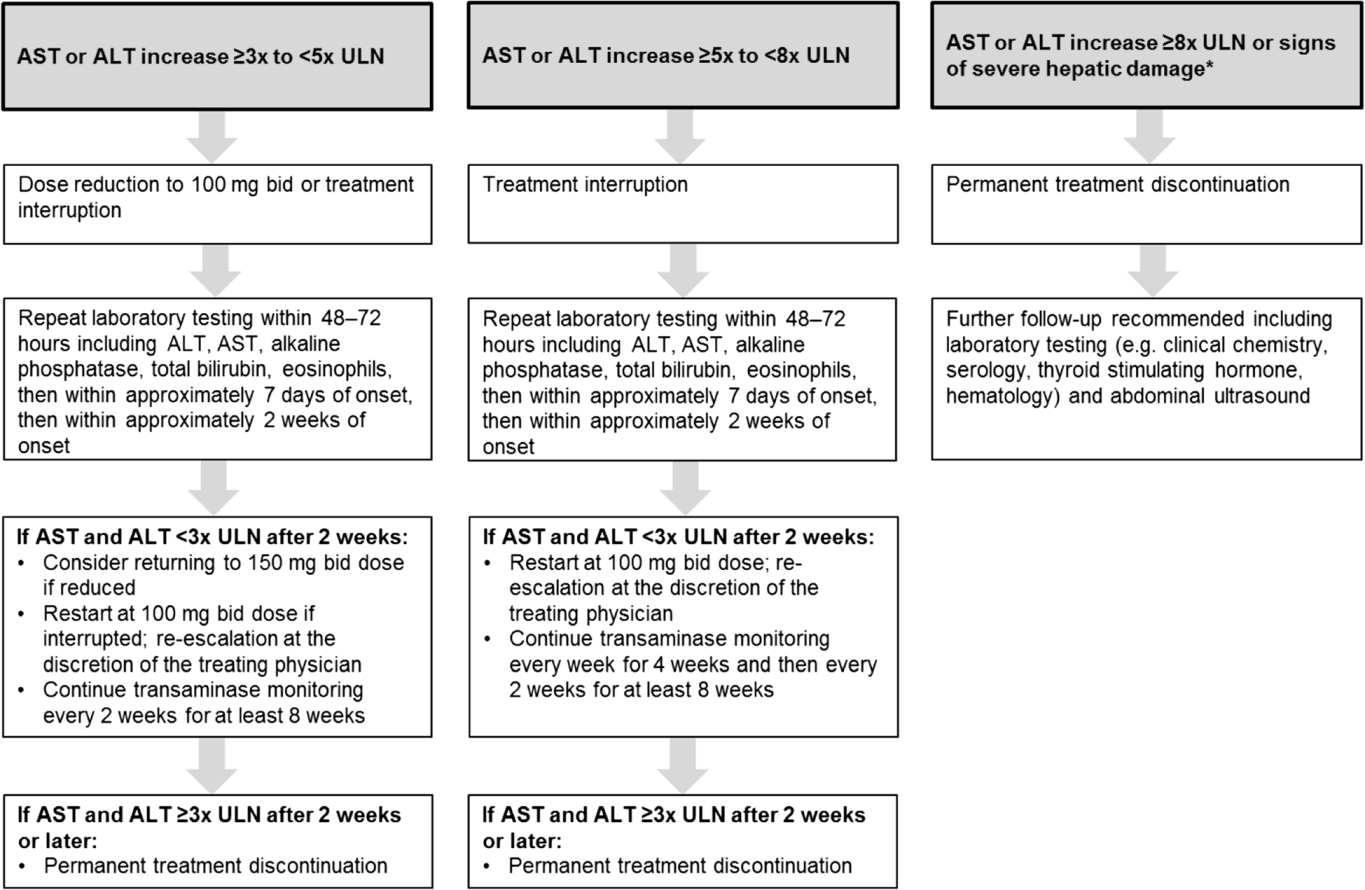
Algorithm for the management of hepatic enzyme elevations in the INPULSIS^®^ trials. *Defined as increase in hepatic transaminases (AST or ALT) to ≥3 x ULN, and i) total bilirubin >1.5 ULN or ii) INR >1.5 or iii) appearance of fatigue, nausea, vomiting, right upper abdominal quadrant pain or tenderness, fever, rash and/or eosinophilia (>5%). In the INPULSIS® trials, routine clinical laboratory testing was undertaken at screening and weeks 2, 4, 6, 12, 18, 24, 30, 36, 44 and 52

Eligible patients were randomized using a 3:2 ratio to receive nintedanib 150 mg bid or placebo for 52 weeks, followed by a 4-week follow-up period. There was no requirement for dose titration on initiation of treatment. Treatment interruption and/or dose reduction from 150 mg bid to 100 mg bid were allowed for the management of adverse events. For patients who had a dose interruption, treatment could be reinstituted at a dose of 150 mg bid or 100 mg bid after resolution of the adverse event. After a dose reduction, the dose could be re-escalated to 150 mg bid. Specific recommendations were provided to the investigators for the management of diarrhea (Fig. 1) and hepatic enzyme elevations (Fig. 2).

For patients who had >1 treatment interruption, the duration of treatment interruption was calculated as total duration of all interruptions. Dose intensity was defined as the amount of drug administered over the study divided by the amount of drug that would have been received had the 150 mg bid dose been administered throughout the 52-week study or until discontinuation in patients who discontinued prematurely. A pre-specified subgroup analysis assessed the impact of dose intensity (≤90 % versus >90 %) on the annual rate of decline in FVC using pooled data from the two trials.

Compliance with study medication was defined as the number of capsules taken divided by the number of capsules that should have been taken over the trial period. Safety was assessed by means of clinical and laboratory evaluation, clinical assessment of vital signs and physical examination at study visits and the recording of adverse events, coded according to the Medical Dictionary for Regulatory Activities (MedDRA) version 16.1. The analyses were descriptive and based on patients who received ≥1 dose of study medication. Patients with adverse events with onset after the first dose and up to 28 days after the last dose of trial medication were included in the analysis. The intensity of adverse events was rated by the investigators as mild (easily tolerated), moderate (enough discomfort to cause interference with usual activity) or severe (incapacitating or causing inability to work or to perform usual activities). For every adverse event, investigators reported whether they considered the event was related to study drug. For patients who experienced ≥1 diarrhea adverse event, the duration of diarrhea adverse events was calculated as the total duration of all the events. A specific diarrhea electronic case report form (eCRF) was introduced during the course of the trials to characterize diarrhea adverse events. Routine clinical laboratory testing was undertaken at screening and weeks 2, 4, 6, 12, 18, 24, 30, 36, 44 and 52.

Both trials were conducted in accordance with the principles of the Declaration of Helsinki and the Harmonised Tripartite Guideline for Good Clinical Practice from the International Conference on Harmonisation and were approved by local authorities. The clinical protocol was approved by an independent ethics committee or institutional review board at each participating center. All patients provided written informed consent before study entry.

## Results

### Patients

A total of 1061 patients were treated in the two INPULSIS^®^ trials, 638 in the nintedanib group and 423 in the placebo group. Demographics and baseline characteristics were comparable between the treatment groups (Table 1 and Additional file 1).

**Table 1:** Baseline demographics and clinical characteristics in the INPULSIS^®^ trials

	Nintedanib	Placebo
	(*n* = 638)	(*n* = 423)
Male, n (%)	507 (79.5)	334 (79.0)
Age, years, mean (SD)	66.6 (8.1)	67.0 (7.9)
Weight, kg, mean (SD)	79.2 (16.6)	78.6 (16.5)
Race, n (%)		
White	360 (56.4)	248 (58.6)
Asian	194 (30.4)	128 (30.3)
Black	2 (0.3)	0 (0.0)
Missing^a^	82 (12.9)	47 (11.1)
Smoking status, n (%)		
Never smoked	174 (27.3)	122 (28.8)
Ex-smoker	435 (68.2)	283 (66.9)
Current smoker	29 (4.5)	18 (4.3)
Time since diagnosis of IPF, years, mean (SD)^b^	1.7 (1.4)	1.6 (1.3)
FVC, mL, mean (SD)	2714 (757)	2728 (810)
FVC, % predicted, mean (SD)	79.7 (17.6)	79.3 (18.2)
FEV_1_/FVC ratio, %, mean (SD)	81.7 (5.8)	81.7 (6.0)
DL_CO_, % predicted, mean (SD)	47.4 (13.5)	47.0 (13.4)
SpO_2_, %, mean (SD)	95.9 (2.3)	95.8 (2.0)
SGRQ total score, mean (SD)^c^	39.5 (19.2)	39.6 (18.5)

Based on patients who received ≥1 dose of study medication. Data collected at screening unless otherwise stated

^a^It was not permitted to collect data on race in France

^b^At randomization

^c^ *n* = 624 for nintedanib 150 mg bid and *n* = 419 for placebo

### Exposure

The mean (standard deviation [SD]) duration of exposure was 10.3 (3.4) months in the nintedanib group and 10.8 (2.8) months in the placebo group. Compliance with study medication was 96.4 % in the nintedanib group and 96.7 % in the placebo group.

A summary of the proportion of patients requiring dose reductions and treatment interruptions is presented in Table 2. The proportion of patients who had ≥1 dose reduction from 150 mg bid to 100 mg bid was higher in the nintedanib group than in the placebo group (178 patients [27.9 %] versus 16 patients [3.8 %]); 15 patients (2.4 %) in the nintedanib group and none in the placebo group had ≥2 dose reductions from 150 mg bid to 100 mg bid. There was no distinct temporal pattern associated with dose reductions. Forty patients (6.3 %) in the nintedanib group and 7 patients (1.7 %) in the placebo group had ≥1 dose increase from 100 mg bid to 150 mg bid. In total, 76.3 % of patients in the nintedanib group and 97.9 % of patients in the placebo group received 150 mg bid as their last dose.

**Table 2:** Dose reductions and treatment interruptions in the INPULSIS^®^ trials

N (%)	Nintedanib	Placebo
	(*n* = 638)	(*n* = 423)
**Dose reductions**		
Patients with ≥1 dose reduction	178 (27.9)	16 (3.8)
Dose reductions per patient		
1	163 (25.5)	16 (3.8)
2	13 (2.0)	0 (0.0)
> 2	2 (0.3)	0 (0.0)
Time to first dose reduction		
≤ 1 month	20 (3.1)	1 (0.2)
> 1 month to ≤2 months	28 (4.4)	4 (0.9)
> 2 month to ≤3 months	19 (3.0)	0 (0.0)
> 3 month to ≤4 months	18 (2.8)	3 (0.7)
> 4 month to ≤5 months	24 (3.8)	1 (0.2)
> 5 month to ≤6 months	22 (3.4)	1 (0.2)
> 6 months	47 (7.4)	6 (1.4)
Patients with ≥1 dose increase to 150 mg bid	40 (6.3)	7 (1.7)
Patients who took 150 mg bid as last dose	487 (76.3)	414 (97.9)
**Treatment interruptions**		
Patients with ≥1 treatment interruption	151 (23.7)	42 (9.9)
Treatment interruptions per patient		
1	113 (17.7)	39 (9.2)
2	30 (4.7)	3 (0.7)
> 2	8 (1.3)	0 (0.0)
Time to first treatment interruption		
≤ 1 month	32 (5.0)	3 (0.7)
> 1 month to ≤2 months	18 (2.8)	4 (0.9)
> 2 month to ≤3 months	18 (2.8)	6 (1.4)
> 3 month to ≤4 months	21 (3.3)	4 (0.9)
> 4 month to ≤5 months	18 (2.8)	2 (0.5)
> 5 month to ≤6 months	11 (1.7)	3 (0.7)
> 6 months	33 (5.2)	20 (4.7)
Patients re-introduced at the same dose of 100 mg bid after treatment interruption^a^	20 (13.2)	1 (2.4)
Patients re-introduced at a reduced dose of 100 mg bid after treatment interruption^a^	81 (53.6)	9 (21.4)
Patients re-introduced at the same dose of 150 mg bid after treatment interruption^a^	49 (32.5)	32 (76.2)
Patients re-introduced at an increased dose of 150 mg bid after treatment interruption^a^	1 (0.7)	0 (0.0)

Based on patients who received ≥1 dose of study medication

^a^Dose at last re-introduction

A greater proportion of patients treated with nintedanib had ≥1 treatment interruption than those treated with placebo (23.7 % versus 9.9 %); 38 patients (6.0 %) in the nintedanib group and 3 patients (0.7 %) in the placebo group had ≥2 treatment interruptions. There was no discernible accumulation of dose interruptions during a specific time period during the trials. The mean (SD) duration of interruption was 25.1 (18.8) and 25.6 (15.3) days in the nintedanib and placebo groups, respectively.

In total, 24.5 % of patients in the nintedanib group and 18.9 % in the placebo group prematurely discontinued trial medication; 19.3 % and 13.0 % of patients treated with nintedanib and placebo, respectively, prematurely discontinued trial medication due to an adverse event.

Mean dose intensity was high in both treatment groups. In the subgroup of patients with dose intensity >90 % (*n* = 484), mean (SD) dose intensity was 99.3 (2.2) % in patients treated with nintedanib and 99.8 (1.6) % in patients treated with placebo. In the subgroup of patients with dose intensity ≤90 % (*n* = 154), mean (SD) dose intensity was 76.1 (10.1) % in patients treated with nintedanib and 80.5 (7.5) % in patients treated with placebo. The adjusted annual rate of decline in FVC was of similar magnitude in patients treated with nintedanib with dose intensity >90 % as in patients treated with nintedanib with dose intensity ≤90 % (mean [standard error] 112.7 [12.8] and 72.7 [24.3] ml/year, respectively).

### Adverse events

A summary of adverse events is presented in Table 3. In total, 95.5 % of patients treated with nintedanib and 89.6 % of patients treated with placebo experienced ≥1 adverse event. The proportion of patients who had ≥1 serious adverse event was similar between the nintedanib (30.4 %) and placebo (30.0 %) groups.

**Table 3:** Patients with adverse events in the INPULSIS^®^ trials

N (%)	Nintedanib	Placebo
	(*n* = 638)	(*n* = 423)
Any adverse event(s)	609 (95.5)	379 (89.6)
Most frequent adverse events^a^		
Diarrhea	398 (62.4)	78 (18.4)
Nausea	156 (24.5)	28 (6.6)
Nasopharyngitis	87 (13.6)	68 (16.1)
Cough	85 (13.3)	57 (13.5)
Progression of idiopathic pulmonary fibrosis^b^	64 (10.0)	61 (14.4)
Bronchitis	67 (10.5)	45 (10.6)
Dyspnea	49 (7.7)	48 (11.3)
Decreased appetite	68 (10.7)	24 (5.7)
Vomiting	74 (11.6)	11 (2.6)
Adverse event(s) leading to permanent dose reduction	101 (15.8)	2 (0.5)
Adverse event(s) leading to permanent treatment discontinuation	123 (19.3)	55 (13.0)
Adverse events that most frequently led to permanent treatment discontinuation^c^		
Progression of idiopathic pulmonary fibrosis^b^	13 (2.0)	21 (5.0)
Diarrhea	28 (4.4)	1 (0.2)
Nausea	13 (2.0)	0 (0.0)
Decreased appetite	9 (1.4)	1 (0.2)
Pneumonia	6 (0.9)	1 (0.2)
Weight decreased	6 (0.9)	1 (0.2)
Abdominal pain	5 (0.8)	1 (0.2)
Vomiting	5 (0.8)	0 (0.0)
Asthenia	4 (0.6)	0 (0.0)
Increased alanine aminotransferase	4 (0.6)	0 (0.0)
Drug-related adverse events (as reported by the investigators)	455 (71.3)	120 (28.4)
Severe adverse events^d^	174 (27.3)	99 (23.4)
Serious adverse events^e^	194 (30.4)	127 (30.0)
Fatal adverse event(s)	37 (5.8)	31 (7.3)

Based on patients who received ≥1 dose of study medication

^a^Adverse events reported in >10 % of patients in either treatment group

^b^Corresponds to the MedDRA term ‘IPF’, which included disease worsening and IPF exacerbations

^c^Adverse events leading to permanent treatment discontinuation in >0.5 % of patients in either treatment group, by preferred term

^d^A severe adverse event was related to intensity and was defined as an event that was incapacitating or that caused an inability to work or to perform usual activities

^e^A serious adverse event was defined as any adverse event that resulted in death, was immediately life-threatening, resulted in persistent or clinically significant disability or incapacity, required or prolonged hospitalization, was related to a congenital anomaly or birth defect or was deemed serious for any other reason

**Fig. 3:**
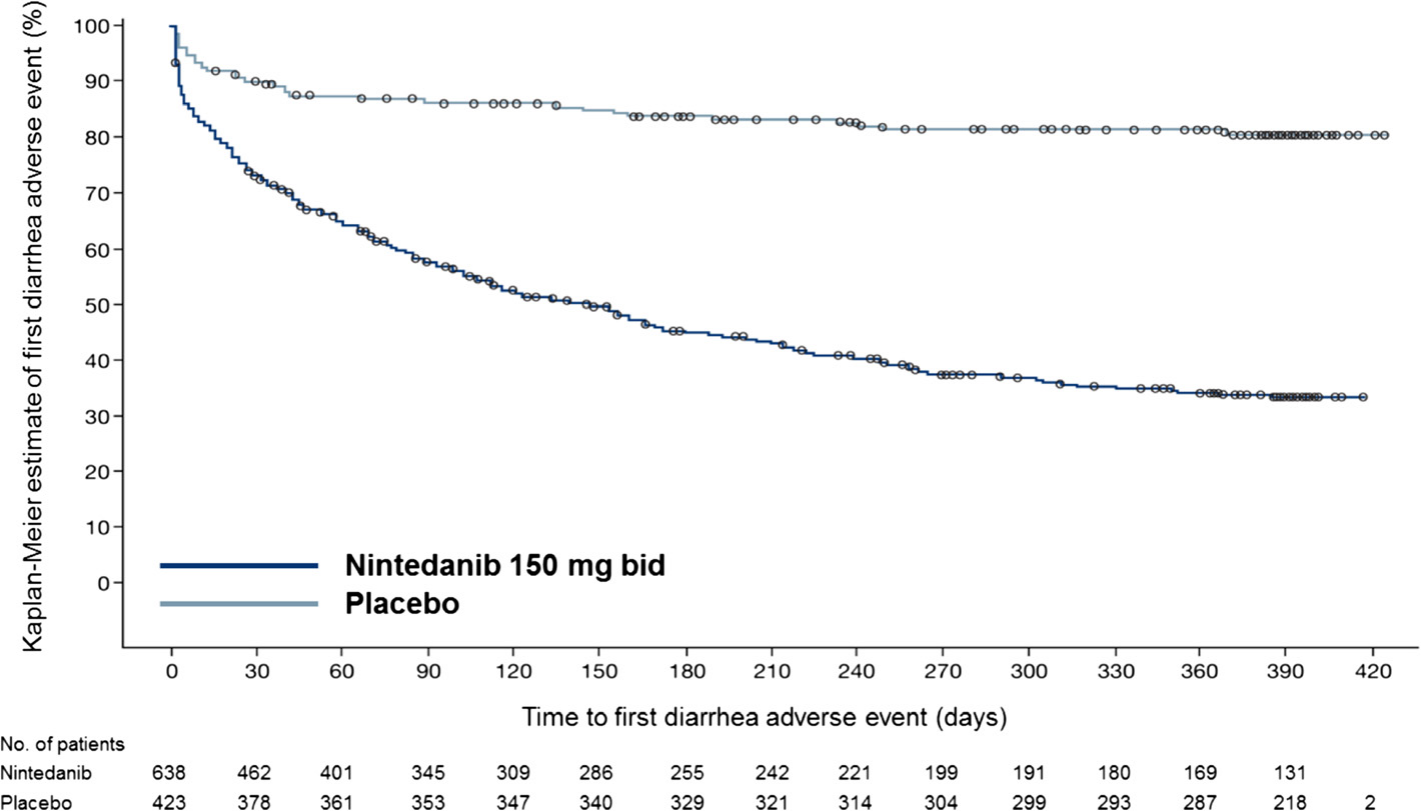
Time to first diarrhea adverse event in the INPULSIS^®^ trials

Diarrhea was the most frequently reported adverse event in the nintedanib group, reported in 62.4 % of patients compared with 18.4 % of patients in the placebo group. A summary of diarrhea adverse events and their clinical consequences is presented in Table 4. Almost all patients who reported diarrhea adverse events (nintedanib: 94.5 %, placebo: 97.4 %) reported events of mild or moderate intensity. Of patients who had ≥1 completed eCRF specific for diarrhea (*n* = 185 in the nintedanib group; *n* = 25 in the placebo group), most had <4 extra stools per day (nintedanib: 111 patients [60.0 %], placebo: 19 patients [76.0 %]). Bowel movements were most commonly characterized as watery with or without formed stool. Among patients who experienced a diarrhea adverse event, anti-diarrheal therapies (most commonly loperamide) were used by 55.3 % of patients in the nintedanib group and 25.6 % of patients in the placebo group during the on-treatment period. For most patients who reported diarrhea adverse events (nintedanib: 78.6 %, placebo: 98.7 %), the events resolved without the need for dose reduction or treatment interruption. A small proportion of patients had a permanent dose reduction (nintedanib: 68 patients [10.7 %], placebo: no patients) and/or discontinued trial medication prematurely due to diarrhea (nintedanib: 28 patients [4.4 %], placebo: 1 [0.2 %]). Among patients who experienced any diarrhea adverse events, most reported a single event (nintedanib: 60.3 %; placebo: 76.9 %) or 2 events (nintedanib: 26.4 %; placebo: 15.4 %). The median (minimum, maximum) duration of diarrhea events was 138.5 (1, 473) days in the nintedanib group and 7.0 (1, 453) days in the placebo group. For most patients with a diarrhea adverse event, the first onset occurred within the first 3 months of treatment (Fig. 3).

**Table 4:** Gastrointestinal adverse events and clinical consequences in the INPULSIS^®^ trials

	Diarrhea		Nausea		Vomiting	
	Nintedanib	Placebo	Nintedanib	Placebo	Nintedanib	Placebo
	(*n* = 398)	(*n* = 78)	(*n* = 156)	(*n* = 28)	(*n* = 74)	(*n* = 11)
Intensity of adverse event^a^						
Mild	226 (56.8)	60 (76.9)	116 (74.4)	26 (92.9)	49 (66.2)	9 (81.8)
Moderate	150 (37.7)	16 (20.5)	38 (24.4)	2 (7.1)	21 (28.4)	2 (18.2)
Severe	21 (5.3)	2 (2.6)	2 (1.3)	0 (0.0)	4 (5.4)	0 (0.0)
Outcome of adverse event^a^						
Recovered	350 (87.9)	72 (92.3)	143 (91.7)	22 (78.6)	69 (93.2)	11 (100.0)
Not yet recovered^b^	43 (10.8)	6 (7.7)	12 (7.7)	6 (21.4)	5 (6.8)	0 (0.0)
Fatal	0 (0.0)	0 (0.0)	0 (0.0)	0 (0.0)	0 (0.0)	0 (0.0)
Unknown	5 (1.3)	0 (0.0)	1 (0.6)	0 (0.0)	0 (0.0)	0 (0.0)
Consequence for dosing^c^						
No permanent dose reduction or discontinuation^d^	313 (78.6)	77 (98.7)	135 (86.5)	28 (100.0)	64 (86.5)	11 (100.0)
Permanent dose reduction of trial drug	57 (14.3)	0 (0.0)	8 (5.1)	0 (0.0)	5 (6.8)	0 (0.0)
Permanent discontinuation of trial drug	28 (7.0)	1 (1.3)	13 (8.3)	0 (0.0)	5 (6.8)	0 (0.0)
Number of adverse events						
1	240 (60.3)	60 (76.9)	116 (74.4)	27 (96.4)	54 (73.0)	11 (100.0)
2	105 (26.4)	12 (15.4)	31 (19.9)	1 (3.6)	14 (18.9)	0 (0.0)
3	29 (7.3)	3 (3.8)	8 (5.1)	0 (0.0)	4 (5.4)	0 (0.0)
≥ 4	24 (6.0)	3 (3.8)	1 (0.6)	0 (0.0)	2 (2.7)	0 (0.0)
Duration of events, days, median (minimum, maximum)^e^	138.5 (1, 473)	7.0 (1, 453)	44.0 (1, 400)	51.0 (1, 404)	6.0 (1, 390)	1.0 (1, 4)

Data are N (%) of patients with ≥1 diarrhea or nausea or vomiting adverse events unless otherwise stated

^a^For patients with more with one event, the intensity/outcome of the worst event is displayed

^b^The patient has not yet returned to his/her previous health status, continues to be followed for the adverse event, but is expected to recover without sequelae

^c^For patients with more than one event, the last consequence for dosing is displayed

^d^Includes patients with temporary dose reductions or treatment interruptions

^e^For patients who experienced ≥1 adverse event, the duration of adverse events was calculated as the total duration of all the events; the definition of a single event was not specified to the investigators in the trial protocol

In a *post-hoc* analysis, the absolute mean (SD) change from baseline in FVC at week 52 was −67.7 (271.4) ml for nintedanib-treated patients who experienced ≥1 diarrhea adverse event (*n* = 398) and −129.4 (246.0) ml for nintedanib-treated patients without any diarrhea events (*n* = 240). For patients treated with placebo, the absolute mean (SD) change from baseline in FVC at week 52 was −227.7 (283.6) ml for patients who experienced ≥1 diarrhea adverse event (*n* = 78) and −197.3 (294.7) ml for patients without any diarrhea events (*n* = 345).

A summary of nausea and vomiting adverse events is presented in Table 4. Almost all nausea and vomiting adverse events were mild or moderate in intensity. Most patients who experienced nausea reported only 1 event (nintedanib: 74.4 %; placebo: 96.4 %). Similarly, most patients who experienced vomiting reported only 1 event (nintedanib: 54 patients [73.0 %], placebo: 11 patients [100 %]). The majority of nausea adverse events had an early onset; vomiting adverse events occurred at any time relative to the start of treatment.

A summary of the proportions of patients with elevations in hepatic enzymes is shown in Table 5. For ALT, AST and bilirubin, the proportion of patients who had normal values at baseline but maximum values above the ULN during treatment was higher in the nintedanib group than in the placebo group. In the majority of patients, values had returned to values within the normal range by the end of treatment. Nevertheless, a greater proportion of patients in the nintedanib group than in the placebo group had a last value on treatment above the ULN for ALT (4.4 % versus 3.4 %) and AST (5.0 % versus 1.7 %). While it is difficult to identify a pattern due to the low number of patients with ALT and/or AST elevations ≥3x ULN, the elevations appeared not to occur at a particular time relative to the start of treatment. Thirty-two patients (5.0 %) in the nintedanib group and 3 patients (0.7 %) in the placebo group had ALT and/or AST elevations ≥3x ULN. Hy’s law criteria were met in 1 patient (in the placebo group).

**Table 5:** Hepatic enzyme elevation adverse events in the INPULSIS^®^ trials

N (%)	Nintedanib	Placebo
	(*n* = 638)	(*n* = 423)
Elevations in ALT and/or AST		
≥ 3x ULN	32 (5.0)	3 (0.7)
≥ 5x ULN	14 (2.2)	1 (0.2)
≥ 8x ULN	5 (0.8)	1 (0.2)
Elevations in maximum total bilirubin		
≥ 1.5x ULN	15 (2.4)	3 (0.7)
≥ 2x ULN	3 (0.5)	2 (0.5)
Elevations in maximum alkaline phosphatase		
≥ 1.5x ULN	37 (5.8)	4 (0.9)
≥ 2x ULN	17 (2.7)	1 (0.2)
Normal ALT values at baseline but maximum value on treatment > ULN on treatment^a^	169 (27.3)	30 (7.2)
Normal AST values at baseline but maximum value on treatment > ULN^b^	134 (21.4)	22 (5.3)
Normal bilirubin values at baseline but maximum value on treatment > ULN^c^	48 (7.7)	22 (5.3)
Normal alkaline phosphatase values at baseline but maximum value on treatment > ULN^d^	94 (15.3)	28 (6.8)

Based on patients who received ≥1 dose of study medication. Categories are cumulative

^a^nintedanib *n* = 620, placebo *n* = 416; ^b^nintedanib *n* = 625, placebo *n* = 418; ^c^nintedanib *n* = 621, placebo *n* = 413; ^d^nintedanib *n* = 615, placebo *n* = 412

Hy’s law criteria were met in no patients in the nintedanib group and in 1 patient in the placebo group. Hy’s law criteria: AST or ALT ≥3x ULN and total bilirubin ≥2x ULN measured in the same blood sample, and no other reason found to explain the combination of increased hepatic transaminases and bilirubin, such as viral hepatitis A, B or C; pre-existing or acute hepatic disease; or the use of another drug capable of causing the observed injury

With regards to other adverse events of interest, bleeding events were reported in 10.3 % of patients in the nintedanib group and 7.8 % of patients in the placebo group. Epistaxis, which occurred in 26 patients (4.1 %) in the nintedanib group and 13 (3.1 %) in the placebo group and contusion, which occurred in 10 patients (1.6 %) in the nintedanib group and 4 (0.9 %) in the placebo group, were the most frequently reported bleeding events. Serious bleeding events occurred with similar incidences in both treatment groups (8 patients [1.3 %] in the nintedanib group and 6 [1.4 %] in the placebo group. Gastrointestinal perforation was reported in 2 patients (0.3 %) in the nintedanib group and no patients in the placebo group. Hypertension was reported in 33 patients (5.2 %) in the nintedanib group and 17 (4.0 %) in the placebo group.

Cardiac disorder adverse events were reported in 10.0 % of patients treated with nintedanib and 10.6 % of patients treated with placebo (Additional file 2). Serious cardiac disorder adverse events were reported in 5.0 % of patients in the nintedanib group and 5.4 % of patients in the placebo group; fatal cardiac disorders occurred in 3 patients (0.5 %) and 6 patients (1.4 %) in the nintedanib and placebo groups, respectively. The MedDRA category of ‘ischemic heart disease’ was reported in a similar proportion of patients in the nintedanib (4.2 %) and placebo (4.0 %) groups (Additional file 2). Within the MedDRA category of ‘ischemic heart disease’, a higher proportion of patients reported an event in the subcategory ‘myocardial infarction’ in the nintedanib group (17 patients [2.7 %]) compared with the placebo group (5 patients [1.2 %]), but a lower proportion of patients reported an event in the other subcategory, ‘other ischemic heart disease’, in the nintedanib group (11 patients [1.7 %]) than in the placebo group (13 patients ([3.1 %]) (Additional file 2). The MedDRA preferred terms of myocardial infarction or acute myocardial infarction were reported in 10 patients (1.6 %) treated with nintedanib and 2 patients (0.5 %) treated with placebo.

A higher proportion of patients experienced weight loss as an adverse event in the nintedanib group (9.7 %) than in the placebo group (3.5 %). The mean change from baseline in body weight at week 52 was −3.1 kg in the nintedanib group and −1.4 kg in the placebo group. The proportion of patients with infection adverse events was similar in both treatment groups (nintedanib: 56.3 %; placebo: 53.9 %) as was the proportion of patients with adverse events related to respiratory, thoracic and mediastinal disorders (nintedanib: 39.8 %; placebo: 41.8 %).

## Discussion

We report collective safety data for nintedanib from the two Phase III INPULSIS^®^ trials. These data demonstrate that nintedanib has a manageable safety and tolerability profile in patients with IPF.

In the INPULSIS^®^ trials, the investigators were provided with recommendations for the management of adverse events, including symptomatic measures as well as the option of a flexible dosing regimen. These recommendations provide guidance for the management of adverse events associated with the use of nintedanib in clinical practice. Although a greater proportion of patients in the nintedanib group had a dose reduction or treatment interruption compared with patients in the placebo group, mean dose intensity in the nintedanib group was high. A pre-specified subgroup analysis demonstrated that nintedanib reduced FVC decline both in patients with a dose intensity of ≤90 % and in patients with a dose intensity >90 %, suggesting that dose reductions or treatment interruptions made to manage adverse events had no major impact on the efficacy of nintedanib.

As expected based on data from previous studies in patients with IPF [10] and patients with solid tumors [13, 14], the most frequently reported adverse events in patients treated with nintedanib in the INPULSIS^®^ trials were gastrointestinal in nature, particularly diarrhea, nausea, vomiting and decreased appetite. Almost all such adverse events were mild or moderate in intensity and most resolved without the need for dose reduction or treatment interruption. A limitation of our studies was that the definition of a single diarrhea event was not specified to the investigators in the trial protocol. Thus, for patients with intermittent diarrhea over a prolonged period, some investigators may have reported this as several events and others as a single event, reducing the precision of our estimate of the number and the duration of diarrhea events. Only 4.4 % of patients in the nintedanib group discontinued trial medication prematurely due to diarrhea, suggesting that recommendations for the management of diarrhea, including symptomatic treatment with anti-diarrheal therapies, were successful in minimizing permanent treatment discontinuations. However, only 55.3 % of patients treated with nintedanib who experienced a diarrhea adverse event received symptomatic anti-diarrheal treatment during the on-treatment period, indicating that there is a need to provide adequate information to physicians in clinical practice and patients about the early management of diarrhea to enable patients to continue taking the drug and so receive maximum benefit from nintedanib treatment. This also applies to anti-emetic treatments. It is recommended that patients treated with nintedanib who experience diarrhea should maintain hydration and take antidiarrheal therapy (e.g., loperamide) as soon as symptoms occur. Dose reduction from 150 mg bid to 100 mg bid or temporary treatment interruption can also be considered. If diarrhea persists, nintedanib should be discontinued. The exact mechanism by which tyrosine kinase inhibition causes nausea or diarrhea is unknown; however, VEGF and VEGF receptors (VEGFR) are known to be highly expressed in the endocrine glands, stomach and intestines, suggesting that VEGF inhibition may cause morphometric changes in the bowel mucosa [15, 16]. A *post-hoc* analysis of data from the INPULSIS^®^ trials showed that nintedanib was effective both in patients who had ≥1 diarrhea adverse event and patients who had no diarrhea adverse events, with a lower mean decline in FVC in patients who had ≥1 diarrhea adverse event. Although the limitations of this descriptive analysis do not allow firm conclusions to be drawn regarding the lower mean decline in FVC in patients who had diarrhea, trials investigating the use of tyrosine kinase inhibitors in oncology have reported an association between drug-related adverse events such as rash and diarrhea and efficacy [17, 18].

In addition to diarrhea, adverse events associated with inhibition of VEGF or VEGFR include arterial hypertension, gastrointestinal perforations, thromboembolism and bleeding [19, 20] and therefore these are potential risks of nintedanib treatment. Patients treated with full-dose anticoagulation or at known risk for bleeding were excluded from the INPULSIS^®^ studies. This has led to recommendations stating that patients at known risk for bleeding should be treated with nintedanib only if the anticipated benefit outweighs the potential risk.

The mechanism behind the elevations in hepatic enzymes or bilirubin following treatment with nintedanib is not well understood, but requires laboratory testing before and periodically during treatment (e.g., during regular patient visits). In general, elevations in hepatic enzymes and total bilirubin were uncommon and returned to normal following dose reduction or treatment interruption.

Although cardiac disorders adverse events were balanced between the nintedanib and placebo groups, a higher proportion of patients in the nintedanib groups had myocardial infarctions. Conversely, a lower proportion of patients in the nintedanib groups had other ischemic heart disease. The clinical significance of this finding is unknown, and further observation is needed. However, it should be noted that the prevalence of myocardial infarctions in the INPULSIS^®^ trials was low and below that observed in patients with IPF based on data from two US health claims databases [21].

In conclusion, pooled safety data from the INPULSIS^®^ trials demonstrate that the dosing regimen utilized and the recommendations given for the management of adverse events, including symptomatic measures and hepatic enzyme monitoring, were successful in minimizing permanent treatment discontinuations. Nintedanib had a manageable safety and tolerability profile in patients with IPF. Physicians, nurses and patients will benefit from clear instructions on how to monitor patients and manage adverse events that may be associated with treatment with nintedanib, consistent with those implemented in the INPULSIS^®^ trials.

## Additional files

Additional file 1:
**Baseline conditions and therapies in the INPULSIS** ^®^ **trials.** (DOCX 15 kb) Additional file 2:
**Cardiac disorders and ischaemic heart disease in the INPULSIS** ^®^ **trials.** (DOCX 14 kb)
