# Supplementary material for: Safety, tolerability and appropriate use of nintedanib in idiopathic pulmonary fibrosis
Source: Respir Res. 2015 Sep 24;16:116. doi: 10.1186/s12931-015-0276-5 (PMC4581488; doi:10.1186/s12931-015-0276-5)
Supplement: Additional file 1: — Baseline conditions and therapies in the INPULSIS ® trials. (DOCX 15 kb) [file 12931_2015_276_MOESM1_ESM.pdf]

Safety, tolerability and appropriate use of nintedanib in idiopathic pulmonary fibrosis

Additional files

Additional file 1

Baseline conditions and therapies in the INPULSIS® trials

Table

	**Nintedanib (n = 638)**	**Placebo (n = 423)**
Concomitant condition(s), n (%)	605 (94.8)	388 (91.7)
Most frequent concomitant conditions, n (%)*		
Hypertension	276 (43.3)	174 (41.1)
Gastresophageal reflux disease	147 (23.0)	101 (23.9)
Hypercholesterolemia	87 (13.6)	71 (16.8)
Diabetes mellitus	88 (13.8)	49 (11.6)
Osteoarthritis	73 (11.4)	52 (12.3)
Hyperlipidemia	86 (13.5)	38 (9.0)
Benign prostatic hyperplasia	54 (8.5)	45 (10.6)
Coronary artery disease	45 (7.1)	43 (10.2)
Treatment with ≥1 therapy, n (%)	567 (88.9)	359 (84.9)
Most frequent therapies, n (%)[†]		
P-Glycoprotein inhibitor[‡]	313 (49.1)	188 (44.4)
Proton pump inhibitor and H2-receptor antagonist	244 (38.2)	162 (38.3)
Anti-platelet[§]	189 (29.6)	118 (27.9)
Systemic corticoid[‖]	136 (21.3)	89 (21.0)
Bronchodilator	129 (20.2)	72 (17.0)

*Concomitant conditions reported in >10% of patients in either treatment group at baseline, by preferred term.

[†]Relevant therapies used by >10% of patients in either treatment group at baseline, by special search category using the World Health Organisation Drug Dictionary.

[‡]Special search category ‘P-Glycoprotein inhibitor’ includes therapies such as omeprazole, simvastatin and atorvastatin.

[§]Prophylactic use of anti-platelet therapy was permitted (e.g., acetylsalicylic acid ≤325 mg/day, clopidogrel 75 mg/day, or equivalent doses of other therapies).
[‖]Concomitant therapy with prednisone ≤15 mg/day, or the equivalent, was permitted if the dose had been stable for ≥8 weeks before screening.
