# Supplementary material for: Safety, tolerability and appropriate use of nintedanib in idiopathic pulmonary fibrosis
Source: Respir Res. 2015 Sep 24;16:116. doi: 10.1186/s12931-015-0276-5 (PMC4581488; doi:10.1186/s12931-015-0276-5)
Supplement: Additional file 2: — Cardiac disorders and ischaemic heart disease in the INPULSIS ® trials. (DOCX 14 kb) [file 12931_2015_276_MOESM2_ESM.pdf]

Safety, tolerability and appropriate use of nintedanib in idiopathic pulmonary fibrosis

Additional files

Additional file 2

Cardiac disorders and ischemic heart disease in the INPULSIS® trials

Table

**N (%)**	**Nintedanib (n = 638)**	**Placebo (n = 423)**
Cardiac disorder adverse events	64 (10.0)	45 (10.6)
Serious cardiac disorder adverse events	32 (5.0)	23 (5.4)
Fatal cardiac disorders	3 (0.5)	6 (1.4)
Ischemic heart disease	27 (4.2)	17 (4.0)
Myocardial infarction	17 (2.7)	5 (1.2)
Myocardial infarction	7 (1.1)	2 (0.5)
Acute myocardial infarction	3 (0.5)	0 (0.0)
Blood creatine phosphokinase increase	5 (0.8)	3 (0.7)
ECG Q-wave abnormal	1 (0.2)	0 (0.0)
Coronary artery occlusion	1 (0.2)	0 (0.0)
Other ischemic heart disease	11 (1.7)	13 (3.1)
Coronary artery disease	5 (0.8)	3 (0.7)
Angina pectoris	2 (0.3)	5 (1.2)
Coronary angioplasty	1 (0.2)	0 (0.0)
Coronary artery stenosis	2 (0.3)	3 (0.7)
Myocardial ischemia	0 (0.0)	3 (0.7)
Coronary artery stent insertion	1 (0.2)	0 (0.0)
ECG ST segment depression	1 (0.2)	0 (0.0)

“Ischemic heart disease” is a standardized MedDRA query (SMQ), comprising the subordinate SMQs “myocardial infarction” and “other ischemic heart disease”.
